# CircPDZD8 promotes gastric cancer progression by regulating CHD9 via sponging miR-197-5p

**DOI:** 10.18632/aging.103805

**Published:** 2020-10-13

**Authors:** Tianfang Xia, Zhenguo Pan, Jie Zhang

**Affiliations:** 1 Department of General Surgery, The Affiliated Huaian No.1 People's Hospital of Nanjing Medical University, Huaian 223300, Huaiyin, Jiangsu Province, China; 2 Department of Gastroenterology, The Affiliated Huaian No.1 People's Hospital of Nanjing Medical University, Huaiyin 223300, Huaian, Jiangsu Province, China

**Keywords:** circRNA, miRNA, ceRNA, gastric cancer

## Abstract

CircRNAs have been shown to be associated with gastric cancer tumorigenesis. But little was known about the role of circPDZD8 in gastric cancer. CircPDZD8 was up-regulated in gastric cancer tissues and cells, Kaplan-Meier survival analysis indicated that gastric patients had a poor overall survival when circPDZD8 levels were high. CircPDZD8 knockdown could hinder proliferation and migration of gastric cancer cells. MiR-197-5p, which was down-regulated in gastric cancer, was shown to be a target of circPDZD8 and was inversely correlated with circPDZD8 expression. CHD9, as a target gene of miR-197-5p, was negatively regulated by miR-197-5p and positively correlated with circPDZD8 expression. Importantly, circPDZD8 could up-regulate CHD9 expression by sponging miR-197-5p, and modulate cell progression by regulation of the miR-197-5p/CHD9 axis in gastric cancer. CircPDZD8 knockdown repressed the progression of gastric cancer cells by sponging miR-197-5p and down-regulating CHD9.

## INTRODUCTION

Gastric cancer is a type of deadly cancer worldwide. The characteristics of high recurrence rate and rapid metastasis of gastric cancer make it more difficult for patients to successfully treat [1–3]. The molecular pathogenesis of gastric cancer is also elusive [4]. Recently, gene therapy for cancer has attracted wide attention and become an important means to study cancer [5–7]. It is an inevitable trend to elucidate the novel gene molecular mechanism related to the development of gastric cancer. Non-coding RNAs were expected to be the star factors in the next generation of molecular biology research [8–10], including long non-coding RNAs (lncRNAs) [11–14], circular RNAs (circRNAs) [15–17] and microRNAs (miRNAs) [18–22]. So far, many studies have proved that lncRNAs are related to the progress of gastric cancer. Xia et al. indicated that circRNA circPDZD8 could promote the progress of gastric cancer [23]. Circular RNA MTO1 suppressed cell growth and motility by modulating miR-3200-5p in gastric cancer [24]. circ_0032821 could predict poor prognosis and augment gastric cancer progress by modulating MEK1/ERK1/2 signaling pathway [25]. CircPDZD8 (hsa_circ_0020123) was first found in gastric cancer [23]. Although, circPDZD8 was upregulated in circRNA microarray, the molecular mechanism of circPDZD8 to regulate gastric cancer progression remains unclear. CircRNAs always may function as competitive endogenous RNA (ceRNA) to sponge miRNA [26], in this study we will study the relationship between circPDZD8 and miRNAs.

MiR-197-5p, as a majority of malignant tumor suppressor gene, has been concerned and studied by most scholars. It was proven that overexpression of miR-197-5p inhibited the sarcomagenesis [27]. MiR-197-5p retarded cell progression via regulation E2F1 in glioma [28]. But the status of miR-197-5p in gastric cancer remains unclear. These results aroused our interest to investigate the effect of miR-197-5p on gastric cancer.

Chromodomain helicase DNA-binding protein 9 (CHD9) belongs to the chromodomain helicase DNA-binding protein family. CHDs are a class of ATP-dependent chromatin modulators that contribute to the chromatin structure reorganization and histone variants deposition [29]. In the CHD family, CHD9 was thought to be an oncogene for clear cell renal cell carcinoma [30] and colorectal cancer [31]. However, whether CHD9 could be modulated by circRNA and miRNA has never been reported in gastric cancer.

The study mainly researched the influence of circPDZD8 on the progression of gastric cancer by modulating miR-197-5p/CHD9 axis, hoping to find novel markers for the diagnosis of gastric cancer.

## RESULTS

### circPDZD8 was upregulated in gastric cancer tissues and cells

**Figure 1:**
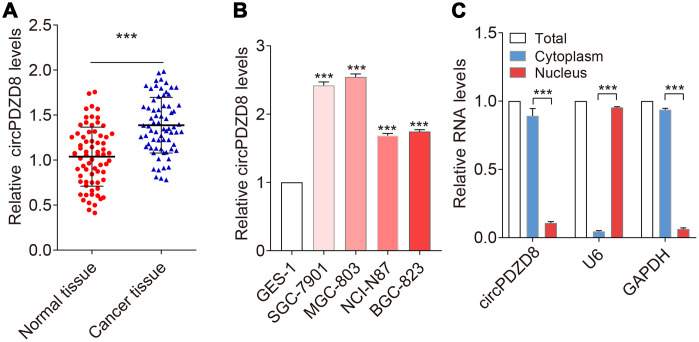
**circPDZD8 was upregulated in gastric cancer tissues and cell lines.** (**A**) circPDZD8 expression was detected in gastric cancer tissues and normal tissues by RT-qPCR (n=20). (**B**) circPDZD8 was upregulated in the different gastric cancer cells. (**C**) circPDZD8 was enriched in MGC-803 cytoplasm fraction. Levels of circPDZD8, GAPDH and U6 RNA in purified MGC-803 nuclear and cytoplasm fractions were detected by RT-qPCR; *** *P* < 0.001.

To explore whether circPDZD8 contributed to the progression of gastric cancer, we first examined the expression level of circPDZD8 in gastric cancer tissues by RT-qPCR. As shown in Figure 1A, circPDZD8 was significantly increased in gastric cancer tissues compared to those in paired normal tissues (n=70). Then, circPDZD8 expression was detected in human gastric cell line GES-1 and human gastric cancer cell lines SGC-7901, MGC-803, NCI-N87 and BGC-823. The results showed that circPDZD8 expression was also drastically enhanced in gastric cancer cell lines (Figure 1B). Moreover, we found that circPDZD8 was enormously enriched in cytoplasm (Figure 1C).

**Figure 2:**
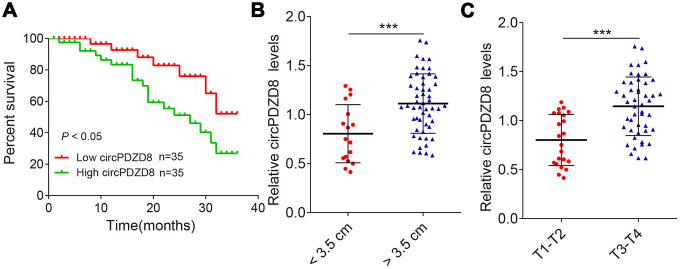
**circPDZD8 negatively correlates with patient prognosis.** (**A**) Kaplan-Meier univariate analysis of overall survival in gastric cancer patients with high (above median) versus low (below median) circPDZD8 levels were shown; *P* < 0.05 [log-rank test]. (**B**) The circPDZD8 was examined in gastric cancer tissues with < 3.5 cm (n = 17) and > 3.5 cm (n = 53). (**C**) The circPDZD8 was examined in gastric cancer tissues in T1-T2 stage (n = 22) and T3-T4 stage (n = 48); *** *P* < 0.001.

Next, the overall survival information was followed up from the patients previously, and the statistical results were analyzed by using the Kaplan-Meier method. It showed that patients who had high levels of circPDZD8 within their gastric cancer tissues had significant shorter overall survival (Figure 2A). Additionally, circPDZD8 was upregulated in gastric cancer tissues that are larger than 3.5 cm (Figure 2B), and also was increased in the group of gastric cancer tissues in advanced stages (Figure 2C), implying the positive association between gastric cancer progression/metastasis and circPDZD8 expression. The data suggested that circPDZD8 might be an oncogene in gastric cancer.

### Silencing circPDZD8 inhibited proliferation and migration of gastric cancer cells *in vitro*

**Figure 3:**
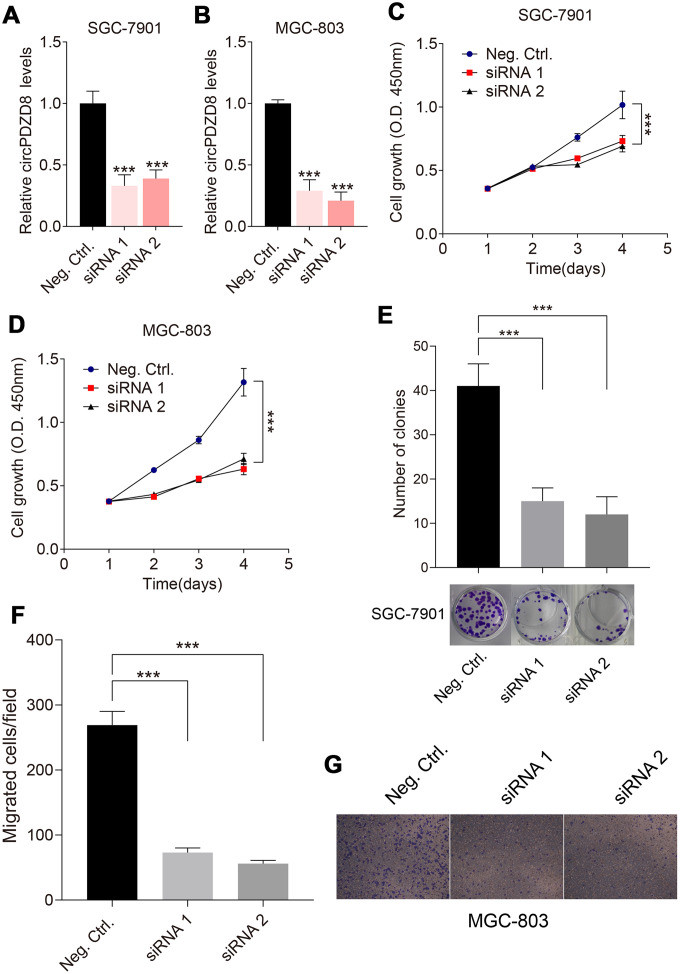
**Effect of circPDZD8 on progress of gastric cancer cells.** (**A**, **B**) circPDZD8 expression in SGC-7901 and MGC-803 cells transfected with si-circPDZD8-1, si-circPDZD8-2 or si-NC was examined by RT-qPCR. (**C**, **D**) The proliferation of SGC-7901 and MGC-803 cells transfected with si-circPDZD8-1, si-circPDZD8-2 or si-NC was measured by MTT assay. (**E**) Clone formation assay demonstrated the clone number in the circPDZD8 knockdown transfection group and the control transfection. (**F**) The migration of SGC-7901 and MGC-803 cells transfected with si-circPDZD8-1, si-circPDZD8-2 or si-NC was examined by Transwell assay without Matrigel. (**G**) Representative images of migrated cells in the Transwell migration assay; *** *P* < 0.001.

Considering the high expression of circPDZD8 in gastric cancer, we examined the effect of circPDZD8 knockdown on the biological behavior of gastric cancer cells. First, we interfered with circPDZD8 in SGC-7901 and MGC-803 cells by transfecting si-circPDZD8. As shown in Figure 3A, 3B, siRNAs targeting circPDZD8 significantly reduced the expression level of circPDZD8 in SGC-7901 and MGC-803 cells. Subsequently, MTT assay demonstrated that circPDZD8 knockdown markedly impaired the proliferation of SGC-7901 and MGC-803 cells (Figure 3C, 3D). Cell colony formation assay indicated that circPDZD8 knockdown inhibited cell colony formation in SGC-7901 cells (Figure 3E). Transwell assay indicated that circPDZD8 knockdown attenuated the migration capacity of SGC-7901 and MGC-803 cells, and significantly decreased the number of migrating cells (Figure 3F, 3G). In short, circPDZD8 knockdown suppressed proliferation and migration of gastric cancer cells *in vitro*.

### circPDZD8 served as a ceRNA for miR-197-5p in gastric cancer cells

**Figure 4:**
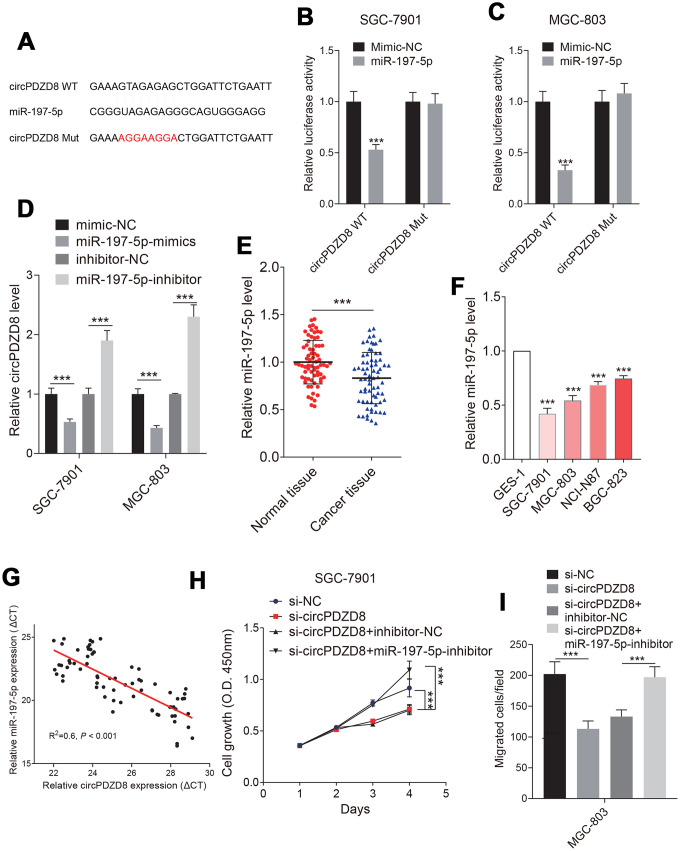
**Studies on the relationship between circPDZD8 and miR-197-5p in gastric cancer cells.** (**A**) The binding sites and mutated sites of circPDZD8 and miR-197-5p were predicted by StarBase v.3.0. (**B**, **C**) The luciferase activity in SGC-7901 and MGC-803 cells co-transfected with circPDZD8-WT or circPDZD8-Mut and miR-197-5p-mimics or mimics-NC was assessed by dual-luciferase reporter assay. (**D**) circPDZD8 expression level in SGC-7901 and MGC-803 cells transfected with miR-197-5p inhibitors or NC were measured by RT-qPCR. (**E**, **F**) miR-197-5p expression in gastric cancer tissues and cell lines was measured by RT-qPCR. (**G**) The correlation of circPDZD8 and miR-197-5p in gastric cancer tissues was analyzed by Pearson’s test (R^2^=0.6, *P* < 0.001). (**H**) The proliferation of SGC-7901 and MGC-803 cells transfected with si-NC, si-circPDZD8-1, si-circPDZD8-1 + inhibitor-NC or si-circPDZD8-1 + miR-197-5p-inhibitor was examined by MTT assay. (**I**) The migration of SGC-7901 and MGC-803 cells transfected with si-NC, si-circPDZD8-1, si-circPDZD8-1 + inhibitor-NC or si-circPDZD8-1 + miR-197-5p-inhibitor was detected by Transwell assay without Matrigel; *** *P* < 0.001.

LncRNAs have been shown to sponge miRNAs and modulate gene expression [32, 33]. To test whether circPDZD8 has a similar mechanism in gastric cancer cells, we used StarBase v.3.0 to predict potential miRNAs that could bind to circPDZD8. As shown in Figure 4A, circPDZD8 and miR-197-5p had potential binding sites and the dual-luciferase reporter assay further demonstrated the targeted relationship between them. The results showed that the luciferase activity in SGC-7901 and MGC-803 cells co-transfected with circPDZD8-WT and miR-197-5p-mimics was obviously declined compared with that in cells co-transfected with circPDZD8-WT and mimics-NC, and there was no distinct change in the luciferase activity of cells transfected with circPDZD8-MUT (Figure 4B, 4C). Simultaneously, we detected the expression of miR-197-5p in SGC-7901 and MGC-803 cells transfected with si-circPDZD8. The data supported that miR-197-5p knockdown steeply augmented the expression of circPDZD8 (Figure 4D). These results further clarified that circPDZD8 could target miR-197-5p. So, we explored the role of miR-197-5p in gastric cancer. RT-qPCR assay showed that miR-197-5p was markedly constrained in gastric cancer tissues and cell lines (Figure 4E, 4F). Furthermore, miR-197-5p expression was negatively associated with the expression of circPDZD8 in gastric cancer tissues (R^2^=-0.6, *P* < 0.001) (Figure 4G). Given the negative regulation of miR-197-5p expression in gastric cancer cells by circPDZD8, we further investigated whether miR-197-5p could in turn modulate the function of circPDZD8 and a series of recovery experiments were carried out. The results identified that miR-197-5p inhibitor could restore the inhibition of si-circPDZD8 on the proliferation and migration of SGC-7901 and MGC-803 cells (Figure 3H, 3I). These findings strongly indicated that circPDZD8 served as a miRNA decoy for miR-197-5p and implicated in the progression of gastric cancer cells by regulating miR-197-5p.

### CHD9 was directly targeted by miR-197-5p in gastric cancer cells

**Figure 5:**
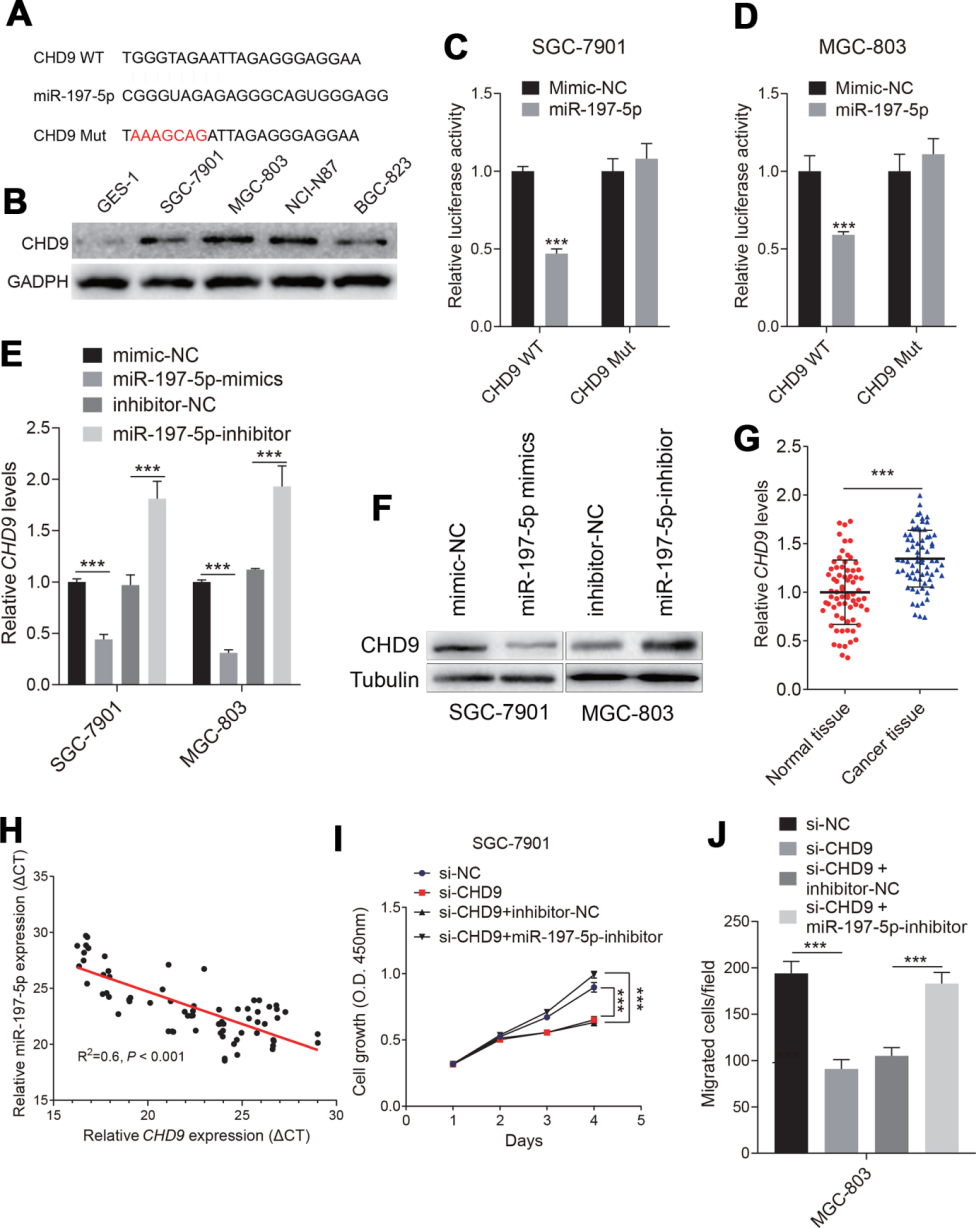
**Identification of the targeting relationship between miR-197-5p and CHD9 in gastric cancer cells.** (**A**) The binding sites of miR-197-5p and mutated CHD9-3’UTR were predicted by StarBase v.3.0. (**B**) The CHD9 expression status in different cell were shown. (**C**, **D**) The luciferase activity in SGC-7901 and MGC-803 cells co-transfected with CHD9-WT or CHD9-MUT and miR-197-5p-mimics or mimics-NC was evaluated by dual-luciferase reporter assay. (**E**, **F**) The mRNA and protein expression levels of CHD9 in SGC-7901 and MGC-803 cells transfected with mimics-NC, miR-197-5p-mimics, inhibitor-NC or miR-197-5p-inhibitor were examined by RT-qPCR and western blot, respectively. (**G**) *CHD9* expression in gastric cancer tissues compared with in normal tissues was detected by RT-qPCR. (**H**) The correlation between miR-197-5p expression and *CHD9* expression in gastric cancer tissues was analyzed by Pearson’s test (R^2^=0.6, *P* < 0.001). (**I**) The proliferation of SGC-7901 and MGC-803 cells transfected with mimics-NC, miR-197-5p-mimics, miR-197-5p-mimics + pcDNA3.1 or miR-197-5p-mimics + pcDNA3.1-CHD9 was examined by MTT assay. (**J**) The migration of SGC-7901 and MGC-803 cells was evaluated by Transwell assay without Matrigel; *** *P* < 0.001.

To further explore the downstream mechanism of circPDZD8/miR-197-5p, we looked for the target genes of miR-197-5p. It was predicted by StarBase v.3.0 that there were binding sites between miR-197-5p and CHD9-3’ UTR (Figure 5A). CHD9 protein levels were increased in gastric cancer cells (Figure 5B). Dual-luciferase reporter assay indicated that miR-197-5p-mimics significantly impaired the luciferase activity in SGC-7901 and MGC-803 cells transfected with CHD9-WT compared with the same cells transfected with CHD9-MUT (Figure 5C, 5D). In addition, the mRNA and protein levels of CHD9 in SGC-7901 and MGC-803 cells were obviously reduced by miR-197-5p-mimics, while miR-197-5p-inhibitor significantly increased the mRNA and protein levels of CHD9 (Figure 5E, 5F). Then we analyzed the expression of CHD9 in gastric cancer tissues and found that CHD9 expression was strikingly upregulated in gastric cancer tissues compared to normal tissues (Figure 5G). What’s more, there was a negatively correlation between the expression of miR-197-5p and CHD9 in gastric cancer tissues (R^2^=-0.6, *P* < 0.001) (Figure 5H). To understand whether CHD9 could affect the regulation of biological behavior in gastric cancer cells by miR-197-5p, CHD9 was overexpressed in SGC-7901 and MGC-803 cells. Knockdown CHD9 significantly inhibited cell growth and migration, while this was reversed when treated with miR-197-5p inhibitor (Figure 5I, 5J). The results of the restoration experiment showed that miR-197-5p-mimics could inhibit the proliferation and migration of SGC-7901 and MGC-803 cells, while the inhibition of miR-197-5p-mimics on cell growth and migration could be recovered by overexpression of CHD9 (Figure 5H, 5I). These results suggested that miR-197-5p directly targeted and regulated CHD9, and CHD9 could regain the inhibition of miR-197-5p on gastric cancer cell progression.

### circPDZD8 upregulated the expression of CHD9 by acting as a ceRNA of miR-197-5p in gastric cancer cells

**Figure 6:**
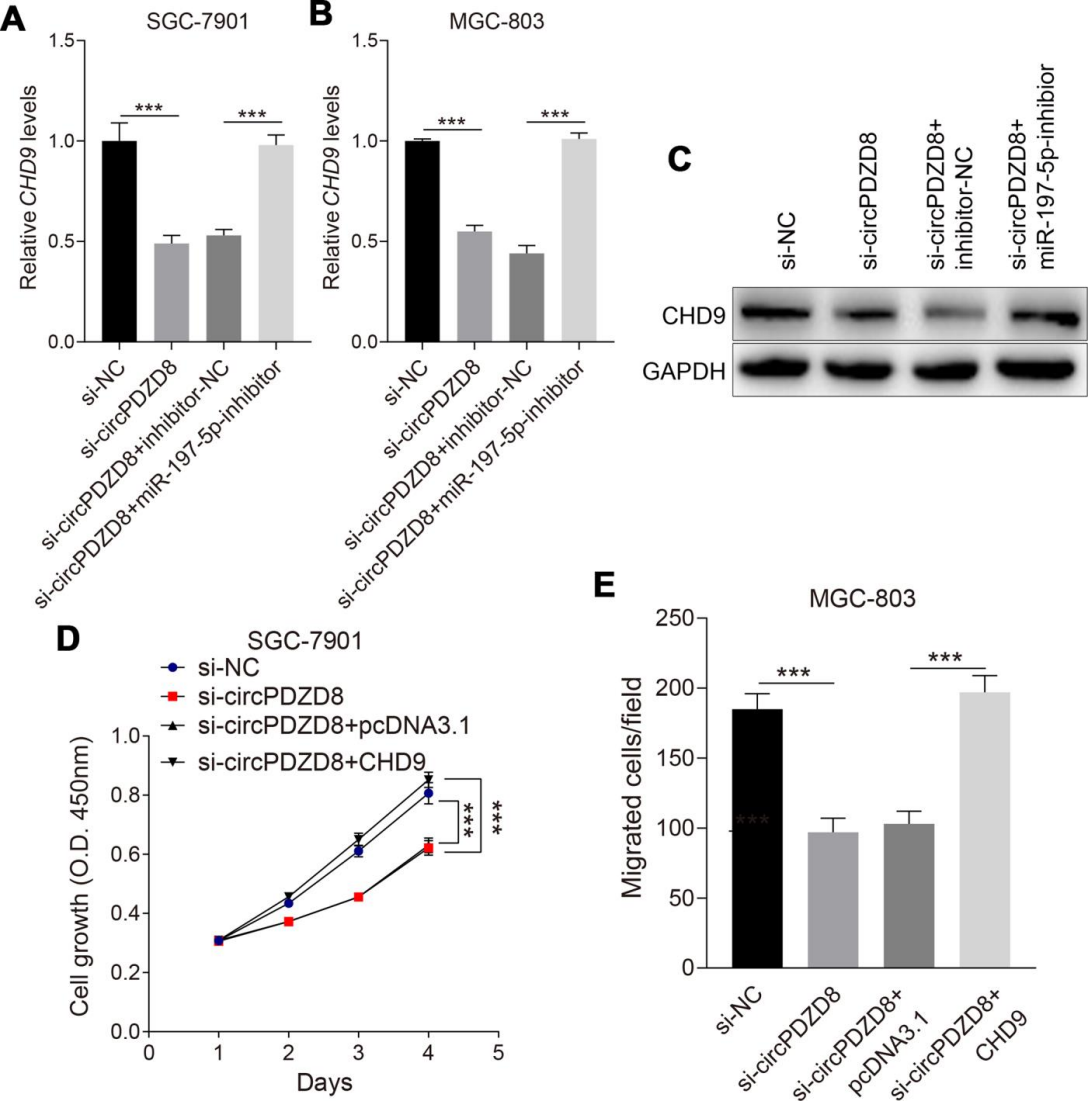
**CircPDZD8 upregulated the expression of CHD9 by modulating miR-197-5p in gastric cancer cells.** (**A**–**C**) The mRNA and protein expression levels of CHD9 in SGC-7901 and MGC-803 cells transfected with si-NC, si-circPDZD8-1, si-circPDZD8-1 + inhibitor-NC or si-circPDZD8-1 + miR-197-5p-inhibitor were measured by RT-qPCR and western blot (MGC-803), respectively. (**D**) The proliferation of SGC-7901 and MGC-803 cells transfected with si-NC, si-circPDZD8-1, si-circPDZD8-1 + pcDNA3.1 or si-circPDZD8-1 + pcDNA3.1-CHD9 was examined by MTT assay. (**E**) The migration of SGC-7901 and MGC-803 cells transfected with si-NC, si-circPDZD8-1, si-circPDZD8-1 + pcDNA3.1 or si-circPDZD8-1 + pcDNA3.1-CHD9 was assessed by Transwell assay without Matrigel; *** *P* < 0.001.

In this part, we aimed to explore whether circPDZD8 could regulate CHD9 expression through miR-197-5p in gastric cancer cells. The data indicated that the declined mRNA and protein expression levels of CHD9 in SGC-7901 and MGC-803 cells caused by si-circPDZD8 could be recovered by co-transfecting with si-circPDZD8 and miR-197-5p-inhibitor (Figure 6A–6C). More than that, overexpression of CHD9 in SGC-7901 and MGC-803 cells could invert the inhibitory effect of si-circPDZD8 on cell proliferation and migration (Figure 6D, 6E). Collectively, these results revealed that circPDZD8 modulated the expression of CHD9 by serving as a ceRNA for miR-197-5p in gastric cancer cells.

### circPDZD8 knockdown inhibited tumor growth by regulating the miR-197-5p/CHD9 axis *in vivo*

**Figure 7:**
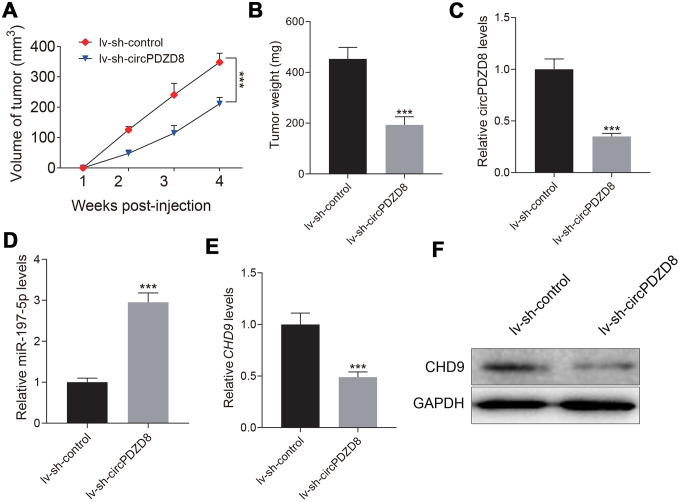
**Effect of circPDZD8 on tumor growth of gastric cancer *in vivo*.** (**A**) Tumor volume was measured at indicated time points after injection of SGC-7901 cells transfected with lv-sh-circPDZD8 or lv-sh-control. (**B**) Tumor weight was measured after the mice were sacrificed. (**C**, **D**) The expression levels of circPDZD8 and miR-197-5p in tumor tissues were measured by RT-qPCR. (**E**, **F**) The mRNA and protein expression levels of CHD9 in tumor tissues were detected by RT-qPCR and western blot, respectively; *** *P* < 0.001.

Next, we established a nude mouse model to study the effect of circPDZD8 on tumor growth *in vivo*. The data showed that tumor growth in nude mice was significantly hindered after subcutaneous inoculation of SGC-7901 cells transfected with lv-sh-circPDZD8, which was reflected by the reduction of tumor volume and weight (Figure 7A, 7B). What’s more, the expression levels of circPDZD8, miR-197-5p and CHD9 in isolated tumor tissues were examined. In the lv-sh-circPDZD8 group, circPDZD8 was obviously declined and miR-197-5p was remarkably elevated compared with that in lv-sh-control group (Figure 7C, 7D). The mRNA and protein levels of CHD9 were significantly decreased in the lv-sh-circPDZD8 group (Figure 7E, 7F). The above results implied that circPDZD8 knockdown could impede tumor growth *in vivo* by modulating the miR-197-5p/CHD9 axis.

## DISCUSSION

circPDZD8 (hsa_circ_0020123) played a pivotal role in the development of non-small cell lung cancer [34]. Qu et al. indicated that circPDZD8 augmented cell proliferation and invasion through suppressing miR-144, resulting in upregulated ZEB1 and EZH2 [34]. These data confirmed that circPDZD8 was positively associated with the proliferation and migration of cancer cells. This positive correlation was due to the high expression of circPDZD8 in cancers. However, the molecular mechanism by which circPDZD8 regulating miRNAs to participate in gastric cancer progression remains unclear. Here, we showed that circPDZD8 was strikingly increased in gastric cancer. Additionally, interference with circPDZD8 impaired proliferation and migration of gastric cancer cells *in vitro*, and retarded the growth of tumor *in vivo*. These results were consistent with previous data [34]. Recently, the competitive endogenous RNAs (ceRNAs) theory revealed a novel regulatory mode of lncRNAs [35]. Wu et al. indicated that circPDZD8 regulated the expression of ACVR1 by competitively binding with miR-384 as a ceRNA in pancreatic cancer [36]. Therefore, we intended to construct an circPDZD8-miRNA-mRNA network to better understand the regulatory effect of circPDZD8 on gastric cancer. First, miR-197-5p was confirmed to be directly targeted by circPDZD8 and their expression in gastric cancer tissues was inversely correlated. In contrast to the circPDZD8, miR-197-5p was significantly downregulated in gastric cancer, which was in accordance with previous results in glioma [28]. More than that, circPDZD8 could negatively regulate miR-197-5p expression in gastric cancer cells, and interfering with miR-197-5p could alleviate the suppressive effects of si-circPDZD8 on the proliferation and migration of gastric cancer cells. These results implied that circPDZD8 induced gastric cancer progression by functioning as a sponge of miR-197-5p. Subsequently, CHD9, a gene targeted by miR-197-5p, attracted our attention. It has been widely reported to be upregulated in many cancers including gastric cancer [30, 31]. In our study, CHD9 was enormously promoted in gastric cancer. Similarly, CHD9 expression in gastric cancer cells was inversely regulated by miR-197-5p and a distinct negative correlation between them was found in gastric cancer tissues. What’s more, up-regulation of CHD9 partially regained the suppressive impacts of miR-197-5p-mimics on proliferation and migration of gastric cancer cells, which was consistent with the previous results that the overexpression of CHD9 could promote the process of gastric cancer cells. To justify the rationality of circPDZD8/miR-197-5p/CHD9 mechanism in gastric cancer, some recovery experiments were carried out. We demonstrated that circPDZD8 not only up-regulated the expression of CHD9 by sponging miR-197-5p but also promoted the progression of gastric cancer cells via modulating CHD9 *in vitro*. And more importantly, knockdown of circPDZD8 could impede the tumor growth via the miR-197-5p/CHD9 axis *in vivo*. These results confirmed our idea that the miR-197-5p/CHD9 regulatory network controlled by circPDZD8 could play a role in the progression of gastric cancer. Overall, we revealed that interference with circPDZD8 inhibited the progression of gastric cancer via modulating CHD9 by competitively binding miR-197-5p. These results implied that circPDZD8 might be a new target for the treatment of gastric cancer as an oncogene.

## MATERIALS AND METHODS

### Clinical tissues and cell culture

90 gastric cancer tissues and matched normal tissues were harvested at The Affiliated Huaian No.1 Hospital of Nanjing Medical University. All patients had not received any treatment before collecting the samples and they had written consent forms. The isolated pieces of tissue were transferred to liquid nitrogen and kept in -80 °C. This study was approved by the ethics committee of The Affiliated Huaian No.1 Hospital of Nanjing Medical University. GES-1 was acquired from American Type Culture Collection (ATCC; Manassas, VA, USA). Human gastric cancer cell lines SGC-7901, MGC-803, BGC-823 and AGS were obtained from The Cell Bank of Type Culture Collection of Chinese Academy of Sciences (Shanghai, China). These cells were grown in Dulbecco Modified Eagle Medium (DMEM) (Sigma-Aldrich, St. Louis, MO, USA) medium with 10% fetal bovine serum (FBS, Sigma-Aldrich) and 1% penicillin-streptomycin at 37 °C and 5% CO2.

### Cell transfection

Small interfering RNA (siRNA) against circPDZD8 (si-circPDZD8), si-NC, miR-197-5p-mimics, mimics-NC, miR-197-5p-inhibitor and inhibitor-NC were synthesized by GenePharma (Shanghai, China). The coding sequence (CDS) of CHD9 was inserted to the pcDNA3.1 vector (Invitrogen, Carlsbad, CA, USA) to enhance CHD9 expression. The lentivirus for circPDZD8 knockdown (lv-sh-circPDZD8) and negative control (lv-sh-control) was purchased from Wuyuan Company (Beijing, China). Lipofectamine 2000 was used for transfection (Invitrogen).

### Quantitative real-time polymerase chain reaction (RT-qPCR)

The RNA of circPDZD8, miR-197-5p or CHD9 was extracted by Trizol reagent (Invitrogen). Reverse transcription kit was purchased from TaKaRa (Wuhan, China). Then, RT-qPCR was performed on ABI 7500 Fast Real-Time PCR system (Applied Biosystems, Carlsbad, CA, USA) using SYBR-Green PCR Master Mix (Thermo Fisher Scientific, Waltham, MA, USA). The levels of circPDZD8 and CHD9 were standardized to glyceraldehyde-3-phosphate dehydrogenase (GAPDH). MiR-197-5p expression was standardized to U6 and calculated according to the 2−Δ ΔCt method. Sequence of primers: circPDZD8, forward 5’-CACTCTGGCCTGCTTC-3’; reverse 5’- CCAGCATACCCATCAGT-3’. GAPDH, forward 5’- CACCCACTCCTCCACCTTTG-3’; reverse 5’-CCACCACCCTGTTGCTGTAG-3’. MiR-197-5p, forward 5’-CCTTCAGCAGCACACTGTGG-3’; reverse 5’- CAGTGCAGGGTCCGAGGTAT-3’. U6, forward 5’-CTCGCTTCGGCAGCACA-3’; reverse 5’-AACGCTTCACGAATTTGCGT-3’. CHD9, forward 5’-ATATTTGTACGCATTCATGTCC-3’; reverse 5’-TACAAAGTCCTAGAAGCACGTT-3’.

### Cell proliferation assay

For the detection of proliferation, SGC-7901, MGC-803 cells were first tiled to 96-well plates for 12 h before transfection. At designated times after transfection, 10 μL of CCK-8 reagent (Dojindo, Kumamoto, Japan) was added into the well and maintained for 3 h. After discarding supernatant, 200 μL of dimethyl sulfoxide (DMSO) was added to solubilize the formazan. Finally, the cell proliferation was examined by measuring the absorbance at 490 nm on a UV microplate reader (Tecan Austria GmbH, Groedig, Austria).

### Transwell migration assay

The migration of SGC-7901, MGC-803 cells was assessed using Transwell assay without Matrigel. Cells were starved for a day before inoculation. Next, Single-cell suspensions were prepared using serum-free medium and transferred to the upper chamber with 100 μL, and 600 μL of DMEM containing 10% FBS was used to fill the lower chamber. One day after treatment, the cells on the lower surface were dyed with 0.1% crystal violet (Sigma-Aldrich) for 20 min and observed using a microscope.

### Dual-luciferase reporter assay

The putative binding sites between circPDZD8 and miR-197-5p as well as between miR-197-5p and CHD9 were predicted by using StarBase v.3.0. Wild circPDZD8 fragment (circPDZD8-WT) containing miR-197-5p binding sites or mutant circPDZD8 fragment (circPDZD8-MUT) without miR-197-5p binding sites were cloned into the pmirGLO vector (EK-bioscience, Shanghai, China). Subsequently, luciferase reporter plasmids were transfected into gastric cancer cells together with miR-197-5p-mimics or mimics-NC. The luciferase activity was measured by a dual-luciferase reporter kit (Solarbio, Beijing, China). In the same way, Wild CHD9-3’UTR fragment (CHD9-WT) or mutant CHD9-3’UTR fragment (CHD9-MUT) containing miR-197-5p binding sites or not was cloned into the pmirGLO vector. The rest of the process was the same as described above.

### Western blot assay

Proteins from SGC-7901, MGC-803 cells and tumor tissues of nude mice were extracted through RIPA reagent (Solarbio). Proteins were then separated and transferred to polyvinylidene difluoride (PVDF) membranes (Thermo Fisher Scientific). After blockage with 5% milk powder at 37 °C for 1 h, the membranes were incubated with the primary antibody of CHD9 (dilution: 1: 1000, Thermo Fisher Scientific) or GAPDH (dilution: 1: 2000, Santa Cruz Biotechnology, CA, USA) at 4 °C for overnight. Then, the membranes were washed and incubated with the secondary antibodies (dilution: 1: 3000, Thermo Fisher Scientific) for 1 h. The membranes were visually detected by the ECL assay (Thermo Fisher Scientific).

### Animal experiment

Four-week-old female nude mice were applied for the study. The animal experiments were permitted by the Animal Ethics Committee of Huaian No.1 Hospital. After transfection with lv-sh-circPDZD8 or lv-sh-control, SGC-7901 cells were injected into the subcutaneous of mice. Tumor volume was surveyed by caliper once a week for 4 weeks. Finally, all the mice were euthanized and tumors were weighed. Relative expression levels of circPDZD8, miR-197-5p and CHD9 in the resected tumors were measured. The body weight and tumor volume [= D × d^2^/2 (mm3), where D is the longest and d is the shortest diameter].

### Statistical analysis

The differences between the two experimental groups were analyzed by Student’s *t* test. Pearson’s correlation coefficient was used to analyze the correlations. Data in this study were expressed as mean ± standard deviation (SD) and repeated at least three times separately. *P* value less than 0.05 was considered statistically significant.
